# Tetrandrine induces muscle atrophy involving ROS-mediated inhibition of Akt and FoxO3

**DOI:** 10.1186/s10020-024-00981-x

**Published:** 2024-11-15

**Authors:** Xin-qi Shan, Na Zhou, Chuang-xin Pei, Xue Lu, Cai-ping Chen, Hua-qun Chen

**Affiliations:** 1 The Jiangsu Key Laboratory for Molecular and Medical Biotechnology, School of Life Sciences, Nanjing Normal University, Nanjing, 210023 China; 2 State Key Laboratory of Natural Medicines, China Pharmaceutical University, Nanjing, 210009 China

**Keywords:** Tetrandrine, Myosin heavy chain, Degradation, ROS, FoxO3/Akt

## Abstract

Tetrandrine (Tet), a well-known drug of calcium channel blocker, has been broadly applied for anti-inflammatory and anti-fibrogenetic therapy. However, due to the functional diversity of ubiquitous calcium channels, potential side-effects may be expected. Our previous report revealed an inhibitory effect of Tet on myogenesis of skeletal muscle. Here, we found that Tet induced protein degradation resulting in the myofibril atrophy. Upon administration with a relative high dose (40 mg/kg) of Tet for 28 days, the mice displayed significantly reduced muscle mass, strength force, and myosin heavy chain (MyHC) protein levels. The MyHC reduction was further detected in C2C12 myotubes after treating with Tet. Interestingly, the expression of Atrogin-1 and Murf-1, the skeletal muscle specific E3 ligases of protein ubiquitin–proteasome system (UPS), was accordingly up-regulated, and the reduced MyHC was significantly mitigated by MG132, a 26S proteasome inhibitor, indicating a key role of UPS in the protein degradation of muscle cells. Further study showed that Tet induced autophagy also participated in the protein degradation. Mechanistically, Tet treatment caused ROS production in myotubes that in turn targeted on FoxO3/AKT signaling, resulting in the activation of UPS and autophagy processes that were involved in the protein degradation. Our study reveals a potential side-effect of Tet on skeletal muscle atrophy, particularly when the drug dose is relatively high.

**Supplementary Information:**

The online version contains supplementary material available at 10.1186/s10020-024-00981-x.

## Introduction

Skeletal muscle serves as the body's primary repository for proteins, accounting for 40% of the body's total weight, and plays a pivotal role in facilitating exercise and energy metabolism (Liang et al. 2020). Muscle loss has a negative impact on daily life, reducing the ability to perform daily activities and prolonging recovery time from disease (Yin et al. 2021). The maintenance of muscle mass predominantly hinges on the delicate equilibrium between protein synthesis and degradation. Disruption in this balance leads to skeletal muscle atrophy showing a loss of muscle mass and strength. It has been well documented that ubiquitination protease system (UPS) elicits protein degradation in a variety of muscle atrophy models (Park et al. 2020), along with up-regulated muscle-specific E3 ligases such as Atrogin-1 and Murf-1 (Bodine et al. 2001; Clarke et al. 2007; Fielitz et al. 2007; Gomes et al. 2001). The autophagy pathway is also considered crucial for the regulation of muscle mass. Under a physiological condition, the basal autophagy levels in skeletal muscle are relatively low. However, it will be elevated significantly under pathological conditions such as oxidative stress and fasting, prompting a significant increase in skeletal muscle autophagy, which leads to degradation of skeletal muscle proteins (Masiero et al. 2009; McGrath et al. 2021; Rocchi and He 2017).

Tetrandrine (Tet), a bisbenzylisoquinoline alkaloid extracted from the root of *S. tetrandra*, has been widely applied in treating various diseases such as tumors, rheumatoid arthritis, and silicosis (Liu et al. 2008; Song et al. 2022; Yuan et al. 2016). Tet exhibited multiple functional properties, such as inhibition of cancer cell proliferation, induction of apoptosis and/or autophagy, inhibiting NLRP3 inflammasome (Song et al. 2022; Huang et al. 2013; Liu et al. 2015a, b; Yu et al. 2016). As a well-known calcium channel blocker, it has long been used in China as an antihypertensive drug (Heister and Poston 2020). Tet drug (Hanfangjijiasu pian, Tetrandrine tablets) has also been clinically applied in silicosis, rheumatic fever, neuralgia and joint pain treatment in China. Recently, three clinical trials of Tet for treatment of SARS-CoV-2 and rheumatoid arthritis are ongoing in China (NCT04308317, Zhejiang; NCT05697029, Beijing; NCT05245448, Beijing). Since calcium channels are widely expressed in human body, Tet may also affect physiological functions of normal cells and lead to side effects. In fact, the toxic effects of Tet have been reported previously and raised safety concerns (Huang and Hong 1998; Mo et al. 2022). Tet at high dosages were toxic in human normal cells, such as MRC-5 human lung cell line, NL-20 human bronchial epithelial cell lines and WI-38 human lung fibroblast (Heister and Poston 2020). We previously identified that Tet at low concentrations (< 2 μmol/L) was non-toxic to skeletal muscle progenitor cells (C2C12 cells and primary myoblasts) but obvious toxic toward myogenic differentiation process of the cell (Li et al. 2022).

In addition to affecting calcium channel, Tet may have effects on multiple other targets and functions in diverse processes. Among those effects, Tet remarkably alters reactive oxygen species (ROS) production in different types of cells (Shen et al. 2010; Wang et al. 2015; Wu et al. 2018). and hence influences mitochondrial function, modulates signal cascades of protein kinase B (Akt) and Forkhead box class O family transcription factors (FoxOs), and other physiological activities (Gumucio and Mendias 2013; Stitt et al. 2004). In the case of skeletal muscle cells, Akt/FoxO3 is capable of modulating the proteolytic system via regulating the expression of ubiquitin–proteasome and autophagy related genes (Sandri et al. 2004; Yin et al. 2021). Whether Tet treatment leads to protein degradation of skeletal muscle remains unknown. To this end, we aim to investigate the effects of Tet on skeletal muscle cells and explore the underlying molecular mechanisms in this study.

## Materials and methods

### Animals

Male C57BL/6 mice (8 weeks) were purchased from GemPharmatech (Nanjing, China). The mice were maintained at the Nanjing Normal University specific-pathogen-free-grade animal facility (Nanjing, China). The animal experiments were approved by the Experimental Committee of Nanjing Normal University (No: IACYUC-20231001). The animals were randomly divided into three groups. Each group of animals (six mice per group) was administered (gavage) with vehicle (0.5% carboxymethyl cellulose) (Sigma-Aldrich, USA) or two doses (20 and 40 mg/kg body weight) of Tet every other day for 28 days. One mouse was dead in 20 mg/kg dosage group due to unknown reason during the study, then there were five mice in this group.

### Cell culture

C2C12 cells which were purchased from ATCC (Rockville, MD, USA) (kindly provided by Prof. Yubo Zhang of the Chinese Academy of Agricultural Sciences, Shenzhen) were cultured in high-glucose Dulbecco’s modified Eagle’s medium (DMEM) (Thermofisher, USA) supplemented with 10% fetal bovine serum (FBS) (Thermofisher, USA) and 1% penicillin and streptomycin (Thermofisher) (Waltham, MA, USA) at 37℃ with 5% CO_2_. For differentiation, when the cell confluence reached approximately 80%, C2C12 myoblasts were switched into differentiation medium (DM, DMEM containing 2% horse serum (Sangon, China) and 1% penicillin and streptomycin (Thermofisher, USA).

### Chemicals and antibodies

Tet [(1b)-6,6′,7,12-tetramethoxy-2,2′-dimethylberbaman] (Chengdu MUST Biotech., China) was dissolved in dimethyl sulfoxide (DMSO) at a concentration of 100 mM as stock solution. NAC (N-acetyl-L-cysteine) was (Beyotime, China) dissolved in PBS at a concentration of 1 M as stock solution. MG-132 (MCE, China) was dissolved in DMSO at a concentration of 10 mM as stock solution.

Myosin heavy chain (MyHC) (MF20) antibody was purchased from DSHB (USA). FoxO3A/p-FoxO3A(Ser253) and GAPDH antibodies were from abcam (China). LC3-I/II, Ubiquitin, P62 and β-actin antibodies were purchased from Sciben (China). Akt/p-Akt (Ser473) and caspase 3/cleaved caspase 3 antibodies were from Cell Signaling Technology (USA). Murf-1 and Atrogin-1 antibodies were from Santa Cruz (USA).

### Western blot analysis

Protein samples were extracted from cells or muscle tissues using radioimmunoprecipitation assay (RIPA) buffer (Beyotime, China) supplemented with a protease inhibitor cocktail (Beyotime, China). Lysates were cleared by centrifugation at 12,000 × *g* (4 ℃, 10 min). The proteins were then separated by sodium dodecyl sulfate–polyacrylamide gel electrophoresis (SDS-PAGE) and transferred onto PVDF membranes (Millipore, USA). Then the membranes were blotted with specific primary antibodies followed by incubation with HRP-conjugated secondary antibodies. The signals were visualized by ECL Chemiluminescent Substrate (Sciben, China) and detected by a Tanon-4500 gel imaging system (China). The intensities of the protein bands were quantified using ImageJ win64 software (USA).

### qRT-PCR analysis

Total RNA was isolated from cells or muscle tissues using TRIzol Reagent (Invitrogen, USA) according to the manufacturer's instructions and cDNA was synthesized using the PrimeScript RT reagent kit (Vazyme, China). Quantitative real-time polymerase chain reaction (qRT-PCR) was carried out using the SYBR Green kit (Vazyme, China). Amplification was performed with the StepOnePlus cycler (Applied Biosystems, USA). Gene expression levels were normalized to the expression of the reference gene GAPDH. The RT-PCR primer sequences were as follows: Atrogin-1: forward primer: 5'-TTCAGCAGCCTGAACTACGA-3'; reverse primer:5'-TCAGCTCCAACAGCCTTACT-3'; Murf-1: forward primer: 5’-GGTGCCTACTTGCTCCTTGT-3’; reverse primer: 5’-CTGGTGGCTATTCTCCTTGGT-3’.

### Measurement of ROS level and mitochondrial membrane potential

DCFH-DA (Sciben, China) staining was used to examine the intracellular ROS level. C2C12 myotubes were incubated with DCFH-DA (10 μM in serum free DMEM medium) for 30 min at 37 °C. The mitochondrial membrane potential of the cells was measured using a JC-1 assay kit (MCE, China). Fluorescent signals were captured under a fluorescence microscopy (Olympus, BX41, Japan).

### Immunofluorescence staining

C2C12 myotubes were fixed in 4% paraformaldehyde (PFA) (Biosharp, China) and permeabilized in 0.3% Triton X-100, blocked with Ready-to-use normal goat serum (Boster, China), and incubated with MF-20 at 4 ℃ overnight, followed by incubation with the secondary antibody. Nuclei were stained with DAPI. Fluorescent images were captured under a fluorescence microscopy (Olympus, BX41, Japan). The diameter of myotubes was measured using Photoshop 2022 software (Adobe Systems Incorporated, USA).

### Grip strength measurement

A grip strength meter (SF-2) (Yibaiyi1B1, China) was used to assess the grip strength of the mice (Li et al. 2021). Mice were lifted by the tail and allowed to grasp a rigid grid attached to a digital force gauge of the strength meter. The tail of each mouse was gently pulled backwards and the tension reading of the digital force gauge was defined as the grip strength before the mouse was released from the grid.

### Wire hanging

In this assay, inverted hanging time was measured. A 2-mm diameter steel wire was placed on a 60–70 cm-high frame above a thick layer of soft bedding (Dorchies et al. 2013). Mice were placed at the center of the wire and the hanging time was recorded until the mouse fell. Each mouse was tested three times with a > 30-min interval between tests, and the average scores were applied to the analysis.

### Histopathological analysis

The muscle tissues were fixed in 4% PFA and embedded in paraffin wax. The sample blocks were sectioned at 5 µm thick, followed by staining with hematoxylin and eosin (H&E) (Beyotime, China). Average cross-sectional area (CSA) of muscle fibers for each condition was quantified using Photoshop 2022 software (Adobe Systems Incorporated, USA) on five chosen fields from the lateral and medial gastrocnemius muscles under a microscope (Olympus, BX41, Japan), with 200 muscle fibers randomly selected for measurement in each field.

### Statistical analysis

Results were presented as mean ± SD. Two-tailed *t* test or one-way ANOVA was used to determine the significance of differences between two groups, followed by Bon ferroni’s post hoc test. All analyses were performed using Graphad prism version 8.0.* p* < 0.05 was considered statistically significant.

## Results

### Tet induces atrophic changes in muscle tissues and myotubes

**Fig. 1:**
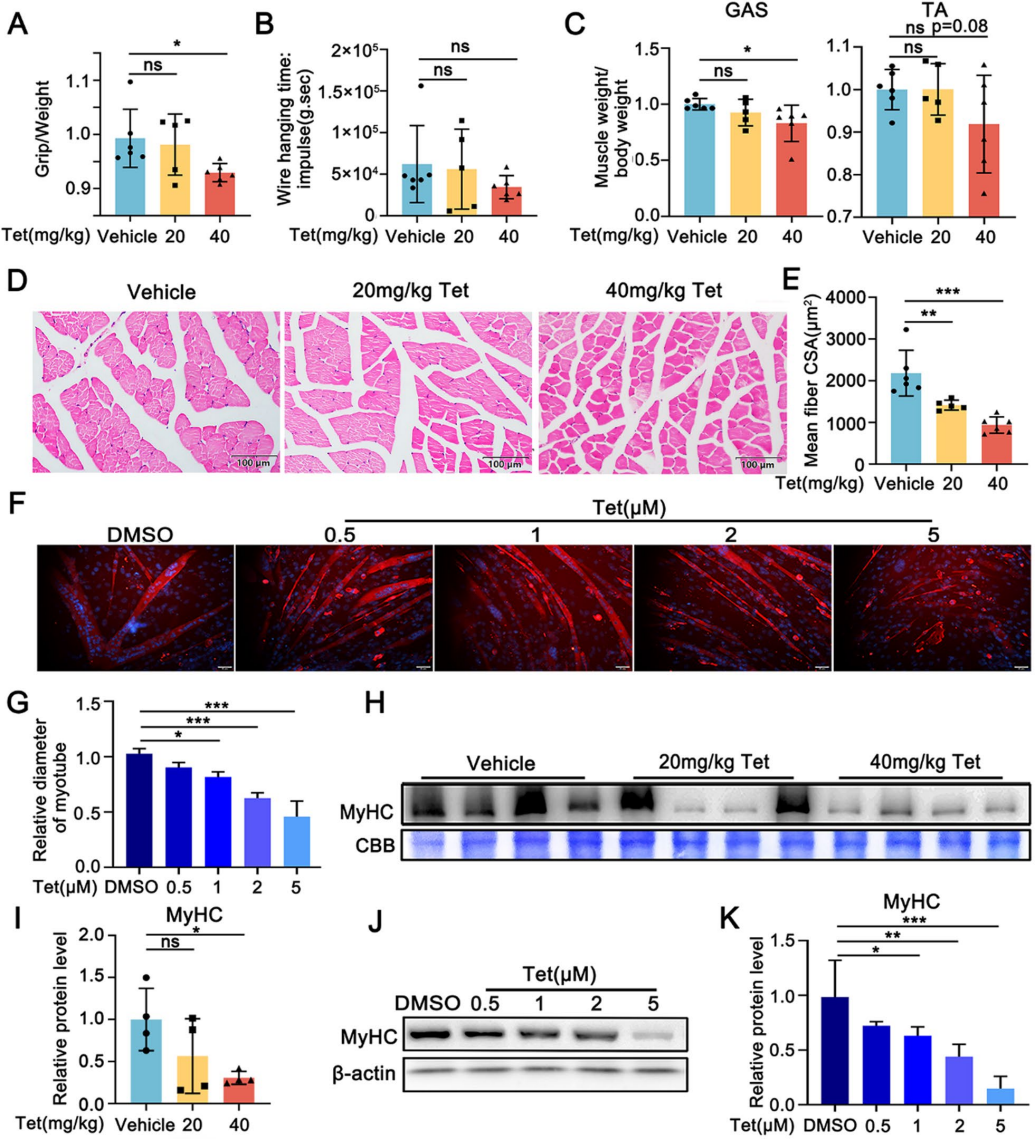
Tet induces muscle atrophy in *vivo* and *vitro*. **A**. The grip strength of Tet treated mice. n = 5–6 per group. **B**. Wire hanging time of Tet treated mice. **C**. The body weight to GAS or TA muscle tissues ratio of Tet treated mice. **D**. H/E staining of cross section of TA muscles. **E**. Quantification of muscle fiber cross-sectional area (CSA) in **D**. Scale bars: 100 μm. **F**. Immuno-staining of Tet treated C2C12 myotubes with MyHC antibody. Scale bars: 50 μm. **G**. Quantification of myotube diameters in **F**. **H**. Western blotting analysis of MyHC protein levels in the GAS muscles of Tet administrated mice. **I.** Quantification of the band intensities in **H**. **J**. Western blotting analysis of MyHC protein levels in Tet treated C2C12 myotubes. **K**. Quantification of the band intensities in **J**. **A-E**: Vehicle and 40 mg/kg: n = 6; 20 mg/kg: n = 5; **F**, **G**, **J** and **K**: n = 3; **H**&**I**: n = 4. Data are shown as mean ± SD. *p < 0.05. **p < 0.01. ***p < 0.001

Firstly, we evaluated the effects of Tet on skeletal muscle mass in vivo. The mice were administrated with Tet by gavage every other day for 28 days and their skeletal muscle contractile functions were examined consecutively. The results showed that both the dosages of 20 mg/kg and 40 mg/kg of Tet caused decline in grip strength and wire hanging time (Fig. 1A–B). A significant decrease in grip strength was detected in 40 mg/kg Tet group compared with the vehicle control. Consistently, the weights of skeletal muscles of Tet administrated mice including GAS and TA muscles were decreased, revealed by the ratio of muscle weights to body weights (Fig. 1C). Histopathological analyses showed that Tet at a higher dosage (40 mg/kg) induced a significant reduction in cross-sectional area (CSA) of the GAS muscle tissues (Fig. 1D–E). Masson’s trichrome staining identified injury of the muscles indicated by apparent fibrosis in the tissues of Tet administrated mice (Fig. S1A&B). However, no obvious morphological changes were examined from the overall view of the GAS muscle sections (Figure S1C). Further study showed that the biochemical marker of the heart, liver and kidney, including serum creatine kinase (CK), aspartate aminotransferase (AST), alanine aminotransferase (ALT), and blood urea nitrogen (BUN), were at relative normal levels (Fig. S2A-D). H/E staining of the sections of these tissues also revealed that no apparent injury was detected in Tet administrated mice (Fig. S2E-J). The above data combined demonstrated that Tet at 40 mg/kg dosage was no toxicity to heart, liver and kidney but skeletal muscle.

We next investigated the effects of Tet on C2C12 myotubes. C2C12 myoblasts were grown in DM for 4 days to induce the formation of myotubes, and then the resultant myotubes were treated with Tet for 3 days. The results showed that Tet (≥ 1 μM) induced a significant decrease in the size of myotubes compared with DMSO control, revealed by the marked reduced diameters of the myotubes (Fig. 1F–G).

Taken together, the results suggested that Tet induced an atrophic morphology of the muscle cells both in vivo and in vitro*.*

The muscle cell atrophic change prompted us to detect the expression of myosin, the most abundant protein in skeletal muscle. By using Western blotting analysis, we observed that the muscle myosin heavy chain (MyHC) protein levels were significantly decreased in 40 mg/kg Tet treated mice compared with vehicle control (Fig. 1H–I). Similar changes of the MyHC protein levels were detected in C2C12 myotubes and primary mouse myoblast differentiated myotubes in the presence of Tet (Fig. 1J–K; Fig. S3).

### UPS plays a key role in Tet induced muscle protein degradation

**Fig. 2:**
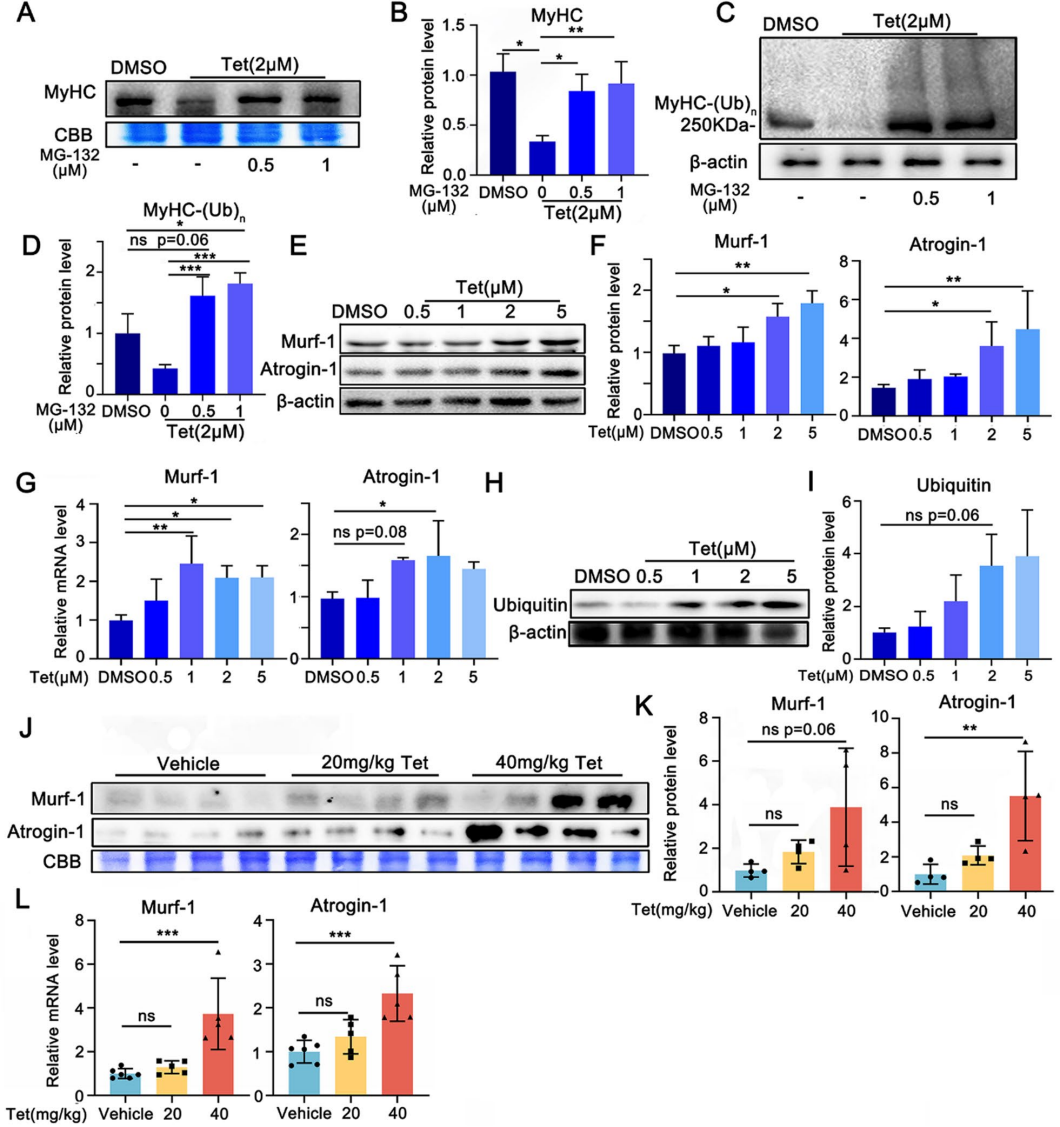
Tet induces UPS activation in muscle cells. **A**. Western blotting analysis of MyHC protein levels in Tet treated C2C12 myotubes in the presence of MG-132. **B**. Quantification of the band intensities in **A**. **C**. Western blotting analysis of poly ubiquitin modified MyHC (MyHC-(Ub)_n_) protein levels in Tet treated C2C12 myotubes in the presence of MG-132. **D**. Quantification of the band intensities in **C**. **E**. Western blotting analysis of Murf-1 and Atrogin-1 protein levels in Tet treated C2C12 myotubes. **F**. Quantification of the band intensities in **E**. **G**. RT q-PCR analysis of mRNA expression levels of Atrogin-1 and Murf-1 in Tet treated C2C12 myotubes. **H**. Western blotting analysis of Ubiquitin proteins levels of Tet treated C2C12 myotubes. **I**. Quantification of the band intensities in **H**. **J**. Western blotting analysis of Murf-1 and Atrogin-1 protein levels in the GAS tissues of Tet administrated mice. **K**. Quantification of the band intensities in **J**. **L**. RT q-PCR analysis of mRNA expression levels of Atrogin-1 and Murf-1 in GAS muscles of Tet administrated mice. **A**–**I**: n = 3; **J**–**K**: n = 4;** L**: n = 5 or 6 as Fig. 1. Data are shown as mean ± SD. *p < 0.05. **p < 0.01. ***p < 0.001

To address whether UPS mediated Tet-induced decrease in MyHC protein, we applied a 26S proteasome inhibitor, MG-132, in the experiments. As shown in Fig. 2A and B, the Tet induced decrease in MyHC protein levels in C2C12 myotubes was greatly mitigated by MG-132 at the concentration of 0.5 μM. Coincide with these results, poly ubiquitin modified MyHC (MyHC-(Ub)_n_) were obvious in Tet treated myotubes in the presence of MG-132, indicating the prevention of 26S proteasome activity dependent degradation of MyHC (Fig. 2C and D).

Given that Atrogin-1 and Murf-1 are two key E3 ligases specifically expressed in the wasting skeletal muscle and mediate the degradation of muscle proteins [4–7], we next measured the expression of these two molecules. As expected, both protein levels were significantly up-regulated in myotubes treated with Tet (Fig. 2E–F), in a dose dependent manner. Consistently, the mRNA levels were also increased (Fig. 2G). In line with the UPS activation, the expression of mono-ubiquitin protein was also increased in the presence of Tet (Fig. 2H-I). The up-regulated Atrogin-1 and Murf-1 expression levels were also detected in skeletal muscles of Tet administered mice at both mRNA and protein levels (Fig. 2J–L). These results indicated that UPS activation played a key role in Tet induced muscle protein degradation.

### Autophagy is involved in Tet induced skeletal muscle cell atrophy

**Fig. 3:**
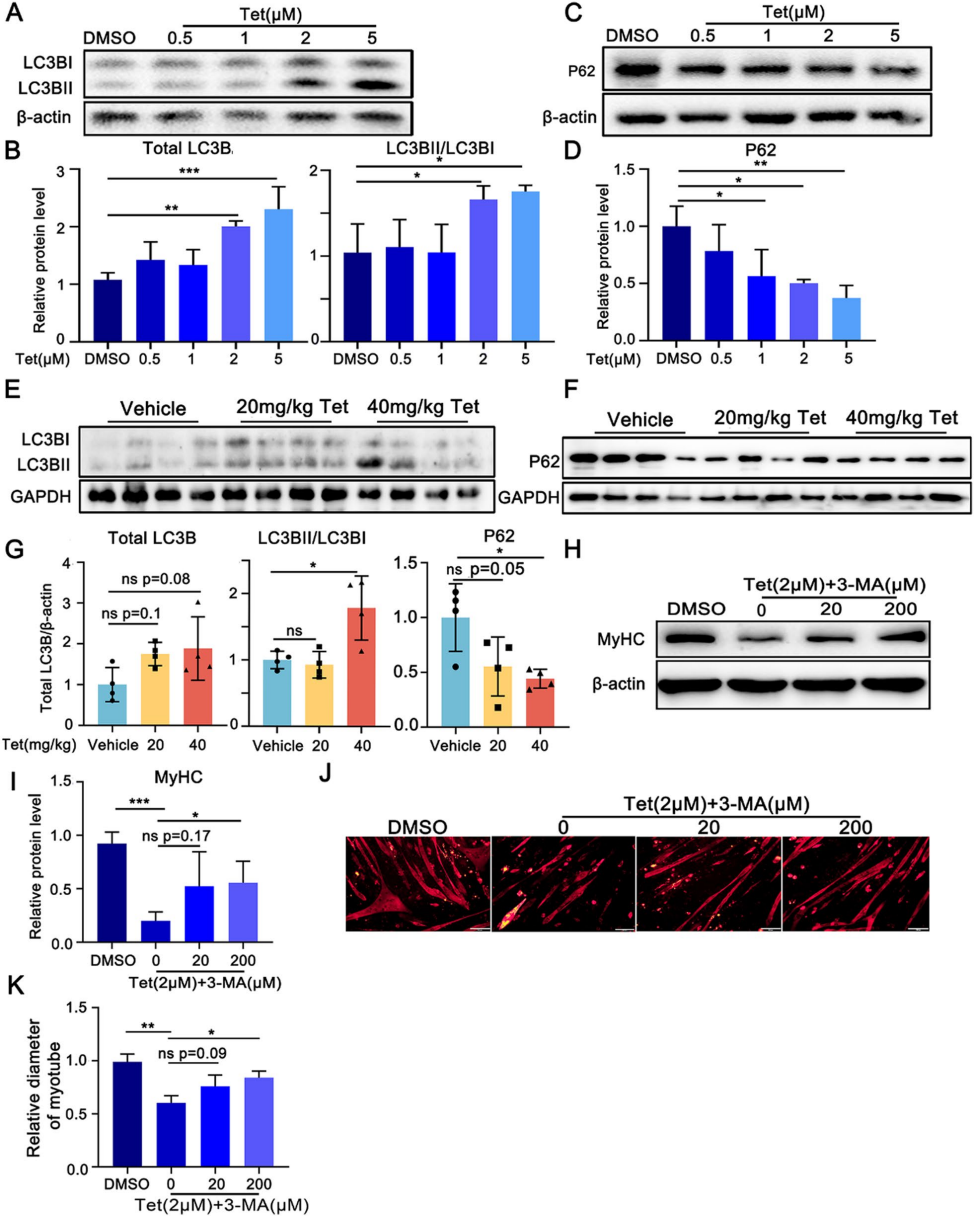
Tet triggers autophagy activation. **A**. Western blotting analysis of LC3B-I and LC3B-II protein levels in Tet treated C2C12 myotubes. **B**. Quantification of the band intensities in **A**. n = 3 per group. **C**. Western blotting analysis of P62 protein levels in Tet treated C2C12 myotubes. **D**. Quantification of the band intensities in **C**. **E**. Western blotting analysis of LC3B-I and LC3B-II protein levels in GAS muscles of Tet administrated mice. **F**. Western blotting analysis of P62 protein levels in GAS muscles of Tet administrated mice. **G**. Quantification of the band intensities in **E** and** F**. **H**. Western blotting analysis of LC3B-I and LC3B-II protein levels in Tet treated C2C12 myotubes in the presence of 3-MA. **I.** Quantification of the band intensities in **G**. **J**. Immuno-staining of C2C12 myotubes with MyHC antibody. Scale bars: 200 μm. **K.** Quantification of myotube diameters in **J**. **A**–**D** & **H**–**K**: n = 3; **E**–**G**: n = 4. Data are shown as mean ± SD. *p < 0.05. **p < 0.01. ***p < 0.001

Autophagy is another protein degradation pathway in skeletal muscle atrophy (Masiero et al. 2009; McGrath et al. 2021). We next evaluated the activation of autophagy in skeletal muscle cells in the presence of Tet. The data showed that this drug apparently caused significant upregulation of total LC3B (LCBI + LC3BII) protein levels and the ratio of LC3BII/I in both the muscle tissues and C2C12 myotubes (Fig. 3A, B, E, G). While the P62 levels were downregulated (Fig. 3C, D, F, G). These data signify the induction of autophagy in Tet treated muscle cells.

We next investigated whether autophagy contributed to Tet induced MyHC degradation by using autophagy inhibitor, 3-Methyladenine (3-MA**)**. Expectedly, 3-MA significantly suppressed Tet induced MyHC reduction in C2C12 myotubes (Fig. 3H–I), and the decrease in myotube sizes was partly attenuated (Fig. 3J–K). These results indicated that autophagy was also involved in Tet induced muscle atrophy.

### Tet induced ROS mediates mitochondria dysfunction and muscle atrophy

**Fig. 4:**
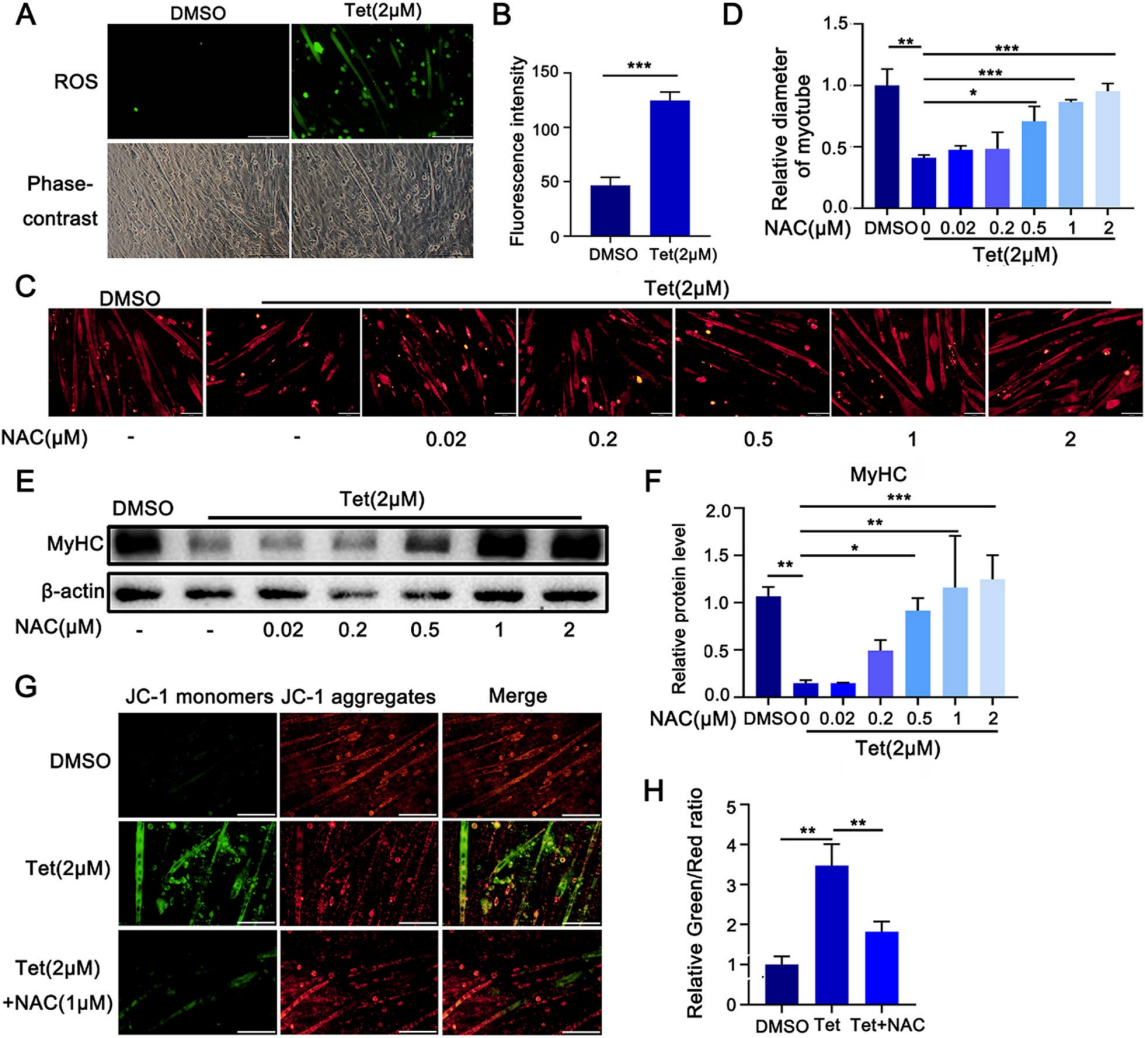
Tet induced ROS production and the effects of ROS in muscle atrophy. **A**. Detection of ROS levels in Tet treated C2C12 myotubes by using of DCFH-DA. Scale bars: 200 μm. **B**. Quantification of the fluorescence intensities in **A**. **C**. Immuno-staining of Tet treated C2C12 myotubes in the presence of NAC. Scale bars: 100 μm. **D**. Quantification of the fluorescence intensities in **C**. **E**. Western blotting analysis of MyHC protein levels in Tet treated C2C12 myotubes in the presence of NAC. **F**. Quantification of the band intensities in **E**. **G**. JC-1 monomers (red) and JC-1 aggregates (green) in C2C12 myotubes. Scale bars: 100 μm. **H**. Quantification of the fluorescence intensities in **G**. n = 3. Data are shown as mean ± SD. *p < 0.05. **p < 0.01. ***p < 0.001

It has been identified that Tet could induce ROS generation and mitochondrial dysfunction in rat hepatocytes (Qi et al. 2013; Yan et al. 2006), we thus measured ROS levels and mitochondria membrane potential in C2C12 myotubes treated with Tet. We found that in contrast to DMSO control, Tet significantly induced ROS production in myotubes at 8 h post incubation (Fig. 4A–B), suggesting a possible role of ROS in Tet-induced muscle atrophy. By using NAC, a commonly used ROS scavenger, the Tet-induced decreases in size of myotubes and MyHC protein levels were significantly attenuated (Fig. 4C–F). In addition, Tet induced mitochondria dysfunction was remarkably restored by NAC pretreatment as evidenced by the alleviated of decreased mitochondrial membrane potential (Fig. 4G–H). These results suggested that Tet-triggered mitochondria dysfunction and atrophic change were associated with ROS generation in muscle cells.

**Fig. 5:**
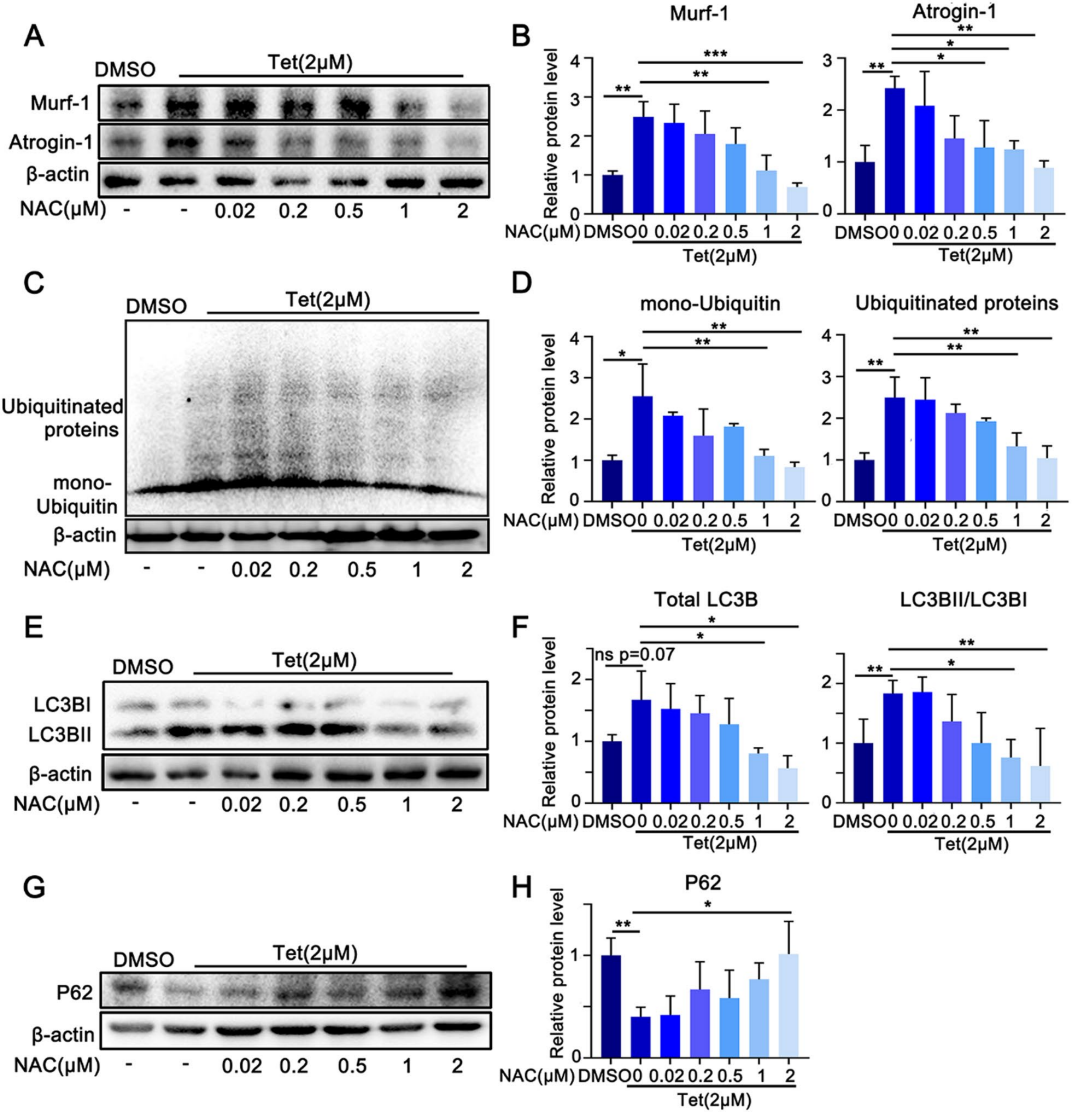
ROS mediates the up-regulation of UPS and autophagy. **A**. Western blotting analysis of Atrogin-1 and Murf-1 expression in Tet treated C2C12 myotubes in the presence of NAC. **B**. Quantification of the band intensities in **A**. **C**. Western blotting analysis of mono-Ubiquitin and Ubiquitinated proteins expression in Tet treated C2C12 myotubes in the presence of NAC. **D**. Quantification of the band intensities in **C**. **E**. Western blotting analysis of LC3B expression in Tet treated C2C12 myotubes in the presence of NAC. **F**. Quantification of the band intensities in **E**. **G**. Western blotting analysis of P62 expression in Tet treated C2C12 myotubes in the presence of NAC. **H**. Quantification of the band intensities in **G**. n = 3. Data are shown as mean ± SD. *p < 0.05. **p < 0.01. ***p < 0.001

We next addressed the relationship between ROS production and upregulation of UPS and autophagy. The myotubes were pretreated with NAC for 1 h, followed by Tet administration for 3 days in the presence of NAC. The results showed that both Tet induced Atrogin-1 and Murf-1 protein increases were dose dependently attenuated by NAC, accompanied by the mitigated increase of mono-ubiquitin and ubiquitin modified proteins (Fig. 5A–D). Moreover, Tet-induced elevation of LC3B was significantly attenuated (Fig. 5E–F). These data indicated that Tet-induced upregulation of UPS and autophagy in muscle cells were associated with ROS accumulation.

### Tet inhibits activation of Akt-FoxO3 axis

**Fig. 6:**
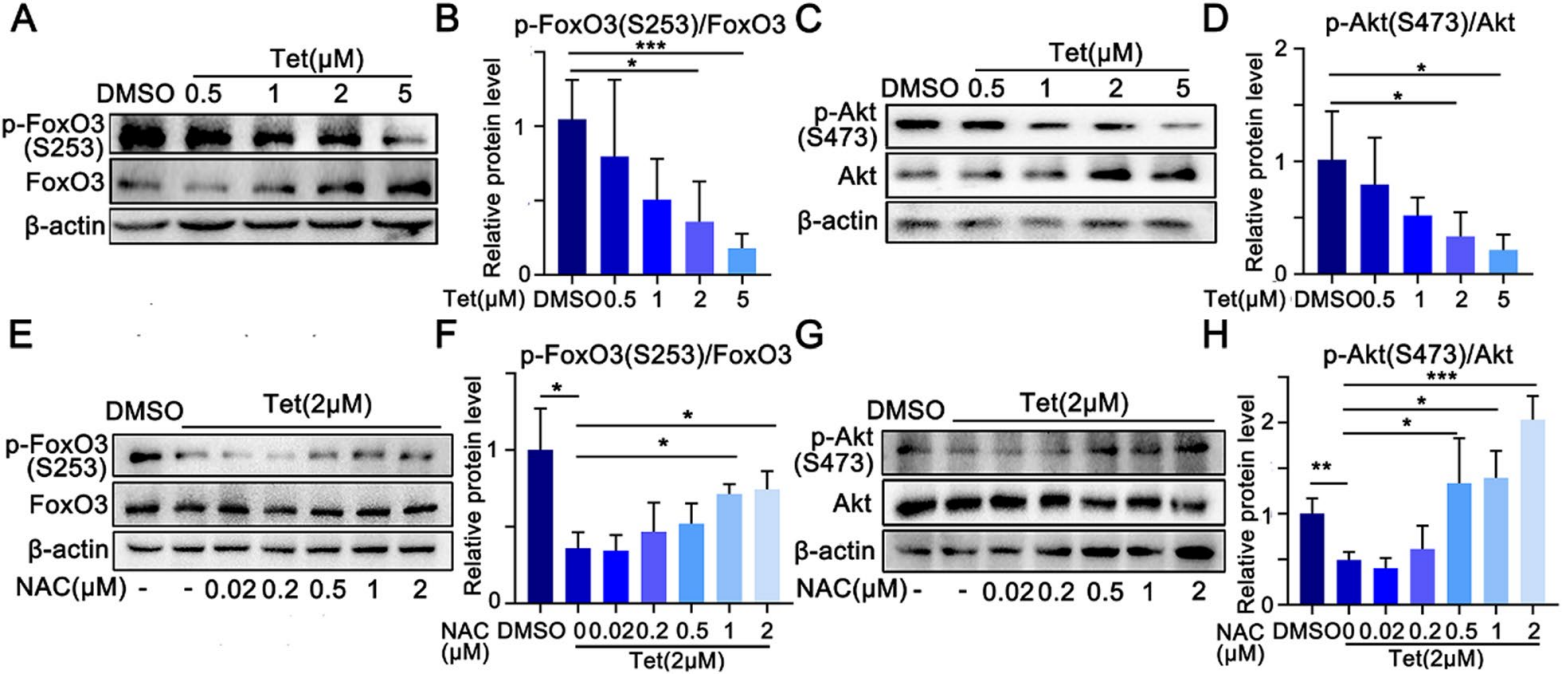
Tet declines Akt and FoxO3 activities. **A**. Western blotting analysis of p-FoxO3(S253)/FoxO3 expression in Tet treated C2C12 myotubes. **B**. Quantification of the ratio of p-FoxO3/FoxO3 in **A**. **C**. Western blotting analysis of p-Akt(S473)/Akt expression in Tet treated C2C12 myotubes. **D**. Quantification of the ratio of p-Akt/Akt in **C**. **E**. Western blotting analysis of p-FoxO3(S253)/FoxO3 expression in Tet treated C2C12 myotubes in the presence of NAC. **F**. Quantification of the ratio of p-FoxO3/FoxO3 in **E**. **G**. Western blotting analysis of p-Akt(S473)/Akt expression in Tet treated C2C12 myotubes in the presence of NAC. **H**. Quantification of the ratio of p-Akt/Akt in **G**. n = 3. Data are shown as mean ± SD. *p < 0.05. **p < 0.01. ***p < 0.001

Previous studies have shown that FoxO3 regulated both UPS and autophagy pathways during muscle atrophy (Milan et al. 2015; Sandri et al. 2004; Yin et al. 2021). Active (phosphorylated) FoxO3 regulates the transcription of target genes including Atrogin-1, MurF-1 and LC3B (Mammucari et al. 2007; Ramaswamy et al. 2002). FoxO3 phosphorylation is primarily regulated by Akt, which keeps this transcription factor in the cytoplasm in an inactive form (Brunet et al. 1999). ROS inhibits activation of Akt (Mo et al. 2022). Given that Tet induced production of ROS in myotubes, we hypothesized that the activation of Akt-FoxO3 signaling would be suppressed by Tet. As expected, both the p-FoxO3 and p-Akt levels were decreased in Tet-treated C2C12 myotubes, in a dose dependent manner (Fig. 6A-D). And these effects were restored by NAC (Fig. 6E–H). Thus, we concluded that ROS initiated Tet-induced skeletal muscle protein degradation through Akt-FoxO3 signaling that in turn activated the UPS and autophagy process.

## Discussion

In this study, we reported that a relative high dose of Tet (40 mg/kg) could induce skeletal muscle atrophy and muscle protein degradation in mice, revealing a side-effect of Tet in body. Similar effects of Tet were observed in C2C12 myotubes and primary myotubes. In vitro study, we demonstrated that Tet-induced muscle protein degradation was correlated to ROS accumulation in C2C12 myotubes, since the ROS scavenger, NAC, clearly attenuated the effects of Tet. Based on our observations, the work flow of Tet in muscle cells can be described below: Tet treatment induced ROS production in intracellular portion, ROS triggered the changes of cellular bio-activities, such as mitochondria dysfunction and reduction of Akt/FoxO3 activities. In turn, the inactivated FoxO3 up-regulated the expression of downstream genes associated with UPS and autophagy, including Atrogin-1, MurF-1 and LC3B, and consequently degrading skeletal muscle protein. Within this process, UPS played a key role.

It has been shown that Tet can modulate cellular activities in a ROS-dependent and independent manner (Mo et al. 2022). In hepatocellular carcinoma cells (HCC), Tet-induced ROS activates autophagy and apoptosis through activating ERK MAP kinase or repressing Akt signaling (Gong et al. 2012; Liu et al. 2011). In leukemia cells, Tet-induced ROS mediates cellular proliferation inhibition, autophagy induction and cell differentiation enhancement (Liu et al. 2015a, b; Wu et al. 2018). Besides cancer cells, Tet could also induce ROS production in normal cells. In rat primary hepatocytes, Tet (10-25 μM) induced ROS led to mitochondria dysfunction and cell apoptosis (Cai et al. 2006). Interestingly, it has been found that Tet induced autophagy in *C. elegans* muscle cells in a ROS-dependent manner (Gong et al. 2012). However, we previously found that in differentiating muscle progenitor cells, Tet inhibited rather than promoted ROS generation, attributing to the blocking of autophagic flux and mitochondria remodeling (Li et al. 2022). Together with the results as we report in this paper, we conclude that the functions of Tet in ROS generation and ROS bioactivity are different, in a cell type dependent manner.

It is worth noting that the altered mitochondrial membrane potential by Tet potentially induces apoptosis of the atrophic muscle cells (N et al. 2019). However, we did not observe elevated caspase 3 activities of the myotubes in the presence of Tet, neither lactate dehydrogenase (LDH) levels (Fig. S4A-C). Thus, apoptosis pathway appears to not be involved in Tet-induced muscle atrophy.

Previous studies showed that low concentrations (≤ 2 μM) of Tet effectively inhibited cell proliferation in cancer cells, while higher concentrations (> 10 μM) of Tet induced cell death (Liu et al. 2017; Qiu et al. 2014; Zhou et al. 2021). In normal cells, the toxic concentration of Tet was much higher than that of cancer cells. The cytotoxic concentration (CC_50_) of Tet was reported to be > 10 μM in an MRC-5 human lung cell line study (Heister and Poston 2020; Kim et al. 2019). Similar results were reported in NL-20 human bronchial epithelial cell lines and WI-38 human lung fibroblast (Heister and Poston 2020; Jin et al. 2011). Another study reported apparent cytotoxicity of high dosages (> 10 μM) of Tet in rat hepatocytes (Qi et al. 2013). Here, we found that Tet induced protein degradation in matured mouse muscle cells at a relative low concentration (1 μM) (Fig. 1J–K), indicating that low dose of Tet could also be toxic in normal cells.

An early toxic study showed that in dogs and monkeys, Tet administrated at 10–150 mg/kg induced severe tissue irritation, lymphoid tissue degeneration and liver damage (Huang and Hong 1998). In literatures, the oral/gavage administrated doses of Tet in mice studies are usually higher than 20 mg/kg, for example, 60 mg/kg for 15 days, 100 mg/kg for 28 days (Liu et al. 2017; Song et al. 2022). The dosage of Tet in humans is usually in the range of 20–150 mg per day depending on the weight and symptoms of the patient. In recent studies on silicosis, Tet was used at an oral dose of 60–100 mg, three times a day, taken for 6 days and stopped for 1 day, with a course of 3 months (Sun et al. 2019). We here found that in mice, 40 mg/kg gavage administered Tet every other day for 28 days is not toxic to liver, heart and kidney (Fig. S1), but induced skeletal muscle atrophy (Fig. 1D-E and Fig. 1H-I). Excitingly, recent study reported that Tet induced hepatotoxicity could be alleviated by Ursolic acid (Xue et al. 2024). Furthermore, many researchers have designed and synthesized new Tet derivatives by modifying its structure to improve their therapeutic properties (Mo et al. 2022; Wei et al. 2016).

In summary, we found that high dose (40 mg/kg) Tet gavage administration caused apparent muscle protein degradation in mouse skeletal muscle along with weakened muscle strength in mice. Ubiquitin–proteasome system and autophagy pathway were involved in the degradation of muscle proteins, which were initiated by Tet-induced ROS accumulation and ROS mediated inactivation of Akt-FoxO3 pathway. Our findings reveal a side-effect of Tet on skeletal muscle, which should be considered during clinical application when relative high dosages of tetrandrine are administrated.

## Supplementary Information

Supplementary material 1.

## Data Availability

No datasets were generated or analysed during the current study.
