# Supplementary material for: Tetrandrine induces muscle atrophy involving ROS-mediated inhibition of Akt and FoxO3
Source: Mol Med. 2024 Nov 15;30:218. doi: 10.1186/s10020-024-00981-x (PMC11566300; doi:10.1186/s10020-024-00981-x)
Supplement: Supplementary file 1 — Supplementary material 1. [file 10020_2024_981_MOESM1_ESM.docx]

**Supplementary methods and materials**

Collagen staining

5 µm cross-sections of GAS muscle tissues were stained with Masson’s trichrome stain kit (Solarbio) (Beijing, China) for detection of collagen fibers. The collagen area from 5 randomly chosen fields for each condition were quantified using ImageJ win64 software (USA).

Assessment of injury markers

Blood samples were collected from the eye sockets to analyze the biochemical markers. The samples were centrifuged at 12,000 rpm, 4°C, for 20 min to separate sera. The serum levels of creatine kinase (CK), aspartate aminotransferase (AST), alanine aminotransferase (ALT) and blood urea nitrogen (BUN) were determined by using the respective kits (Jiancheng) (Nanjing, China) according to the manufacturer's protocols.

Isolation and culture of primary myoblasts

The skeletal muscle tissues dissected from 4 day neonatal mice were cut into small pieces and digested with enzyme mixture (0.75U/mL dispase II (Roche), 1U/mL collagenase D (Roche)) at 37℃. After 0.5 to 1 h, the digested mixture was suspended with Ham's F-10 Nutrient Mixture (Gibco), followed by centrifugation at 1000 rpm for 10 min. The myoblasts were resuspended with growth medium (GM) (half Ham’s F-10 and half high glucose DMEM media containing 20% fetal bovine serum (Gibco), 1% penicillin/streptomycin (Thermofisher) and put in a 24-well plate (Corning). Which was precoated with 0.25% rat tail collagen (Sigma-Aldrich). For differentiation, the myoblasts were switched into differentiation medium DM as C2C12 myoblast.

Lactate dehydrogenase (LDH) assay

Supernatants from Tet treated myotubes were harvested and analyzed for LDH measurements using the Lactate dehydrogenase assay kit (Jiancheng, China) according to the manufacturer’s protocol.

**Supplementary results**

**Figure S1**

**
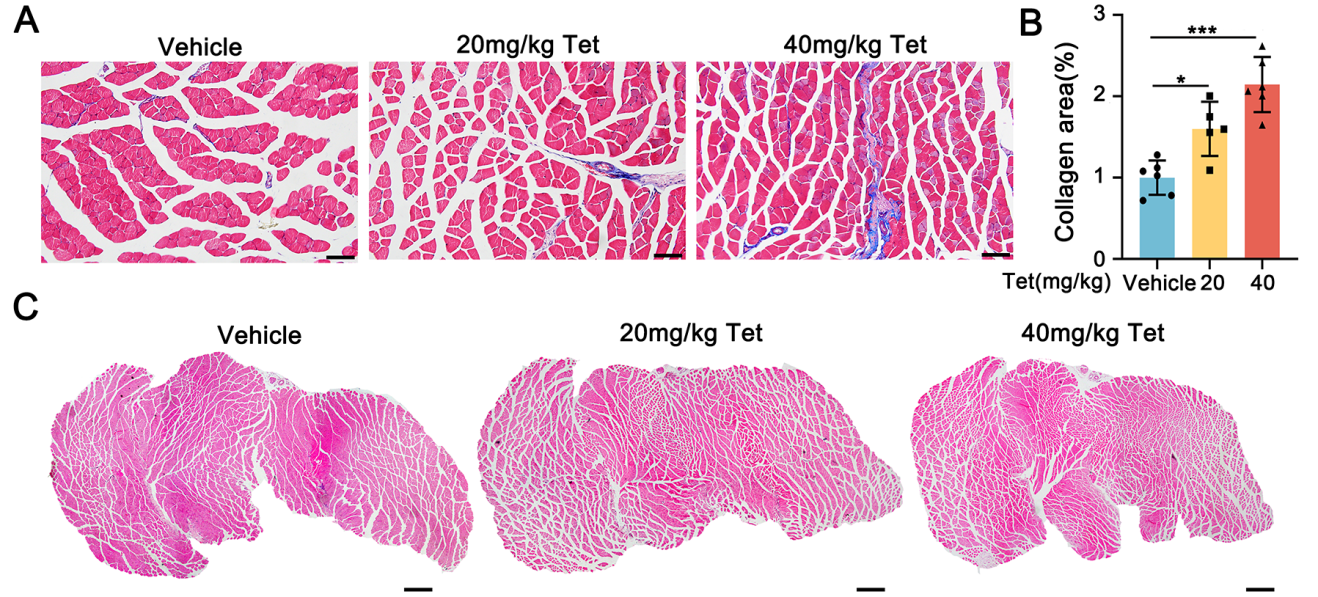
**

**Figure S1** Histopathological analysis of muscle tissues in Tet treated mice. A. Masson’s trichrome-staining of the cross-sections of GAS muscles (blue). Scale bars: 50 µm. B. Relative collagen area in A. Data are shown as mean ± SD. n=6 in vehicle group; n=5 in 20mg/kg Tet group; n=6 in 40mg/kg Tet group. C. Overall view of the sections of GAS muscles with H/E staining. Scale bars: 500 μm.

**Figure S2**


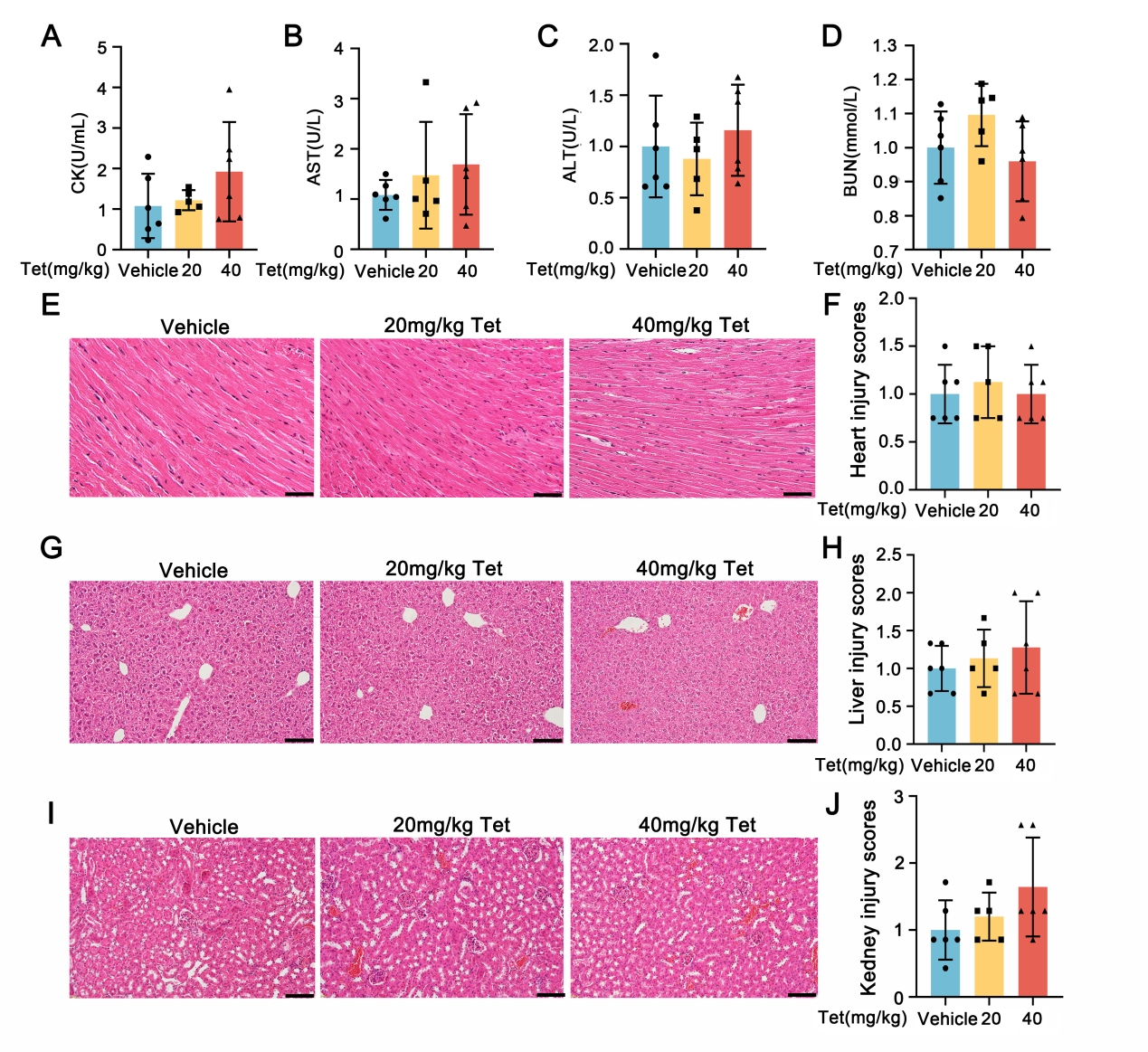


**Figure S2** Effects of Tet on myocardial, liver and kidney. A. The serum CK levels of Tet treated mice. B. The serum AST level of Tet treated mice. C. The serum ALT levels of Tet treated mice. D. The BUN level of Tet treated mice. E. H/E staining of heart. F. The heart injury scores in E. Scale bars: 50 μm. G. H/E staining of liver. H. The liver injury scores in G. n=5-6. Scale bars: 100 μm. I. H/E staining of kidney. J. The kidney injury scores in I. n=5-6. Scale bars: 100 μm. Vehicle control: n=6; 20mg/kg: n=5; 40mg/kg: n=6. Data are shown as mean ± SD.

**Figure S3**

**
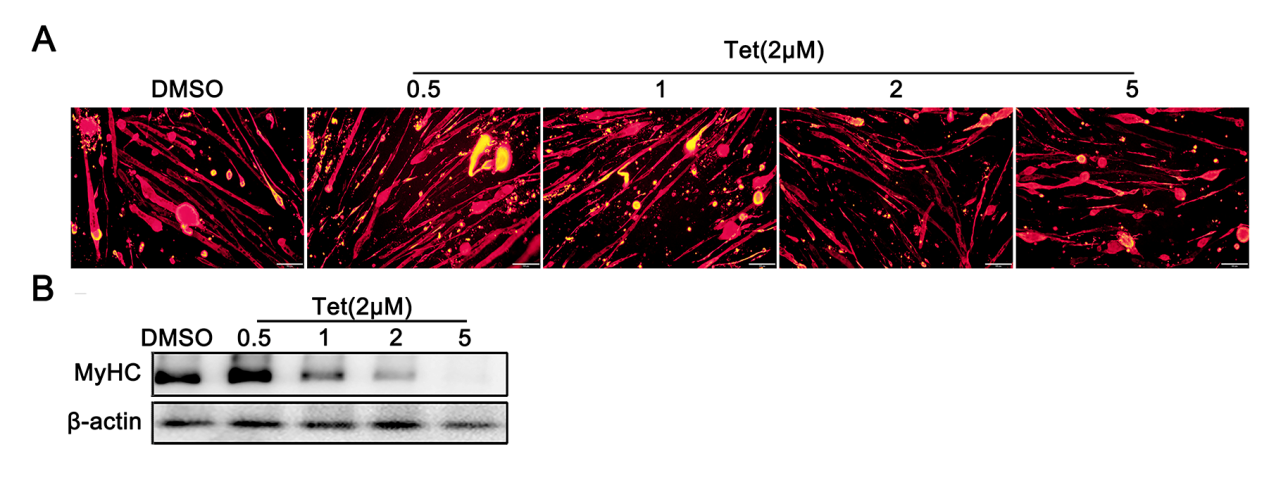
**

**Figure S3** Tet induces atrophy of primary myotubes. a. Immuno-staining of Tet treated primary myotubes with MyHC antibody. Scale bars: 100 μm. b. Western blotting analysis of MyHC protein levels in Tet treated primary myotubes.

**Figure S4**


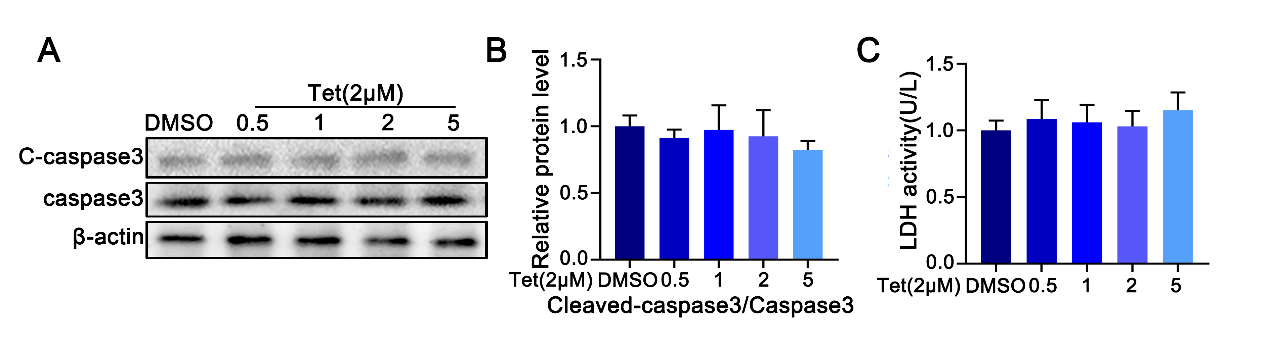


**Figure S4** Caspase 3 activity and LDH levels in Tet treated myotubes. A. Western blotting analysis of Cleaved-caspase3 and Caspase3 protein levels in Tet treated C2C12 myotubes. B. Quantification of the band intensities in A. C. LDH level of Tet treated C2C12 myotubes. Data are shown as mean ± SD. n=3 per group.
